# “JUMPing into Diabetes Control”: A Group-Setting Self-Empowerment Lifestyle Intervention among Diabetes Patients

**DOI:** 10.3390/healthcare8020090

**Published:** 2020-04-07

**Authors:** Sheena Henry, Lu Shi, Virginia Alexander, Richard O’Neal, Stephen Carey, Hugh D. Spitler, Deborah Leonard, Gail Chastain, Lauren Hassan, Meenu Jindal

**Affiliations:** 1 Department of Medicine, University of Missouri, 1 Hospital Dr, Columbia, MO 65212, USA; Henrysh@health.missouri.edu; 2 Department of Public Health Sciences, Clemson University, 507 Edwards Hall, Clemson, SC 29634, USA; hspitle@clemson.edu; 3 Internal Medicine Clinic, Prisma Health, 876 West Faris Rd, Greenville, SC 29605-5601, USA; dralexander@briointernalmedicine.com (V.A.); Deborah.Leonard@prismahealth.org (D.L.); Gail.Chastain@prismahealth.org (G.C.); 4 Department of Medicine, University of Kentucky, 800 Rose Street MN 150, Lexington, KY 40506, USA; Roneal@uky.edu; 5 Atrium Healthcare, 1000 Blythe Blvd. Charlotte, NC 28203, USA; stephencar@pcom.edu; 6 Mayo Clinic, 4500 San Pablo RD S Jacksonville, FL 32224, USA; Hassan.lauren@mayo.edu

**Keywords:** diabetes, group peer support, motivational interviewing

## Abstract

We examined the impact of a group-based self-empowerment intervention among diabetes patients, which uses multidisciplinary education, collaborative learning, peer support, and development of diabetes-specific social capital to improve glycemic control and weight management. Thirty-five patients who had primary care established at the Prisma Health Upstate, Internal Medicine Resident clinic and held the diagnosis of diabetes for longer than one year were recruited for our single-arm pilot intervention. Each group intervention session involved one to two internal medicine resident physician facilitators, a clinical diabetic educator, and 5–10 patients. Each session had a framework facilitated by the resident, with most of the discussion being patient-led, aiming to provide a collaborative learning environment and create a support group atmosphere to encourage self-empowerment. Patients’ hemoglobin A1c level and body mass index (BMI) before the intervention and 3 to 6 months after completion were collected from the laboratory results obtained in the participants’ routine clinic visits. All graduates from this three-week intervention were invited to attend monthly maintenance sessions, and we tracked the HgbA1c measures of 29 JUMP graduates one year after the intervention, even though 13 of the 29 chose not to participate in the monthly maintenance sessions. The pre-intervention HgbA1c level averaged 8.84%, whereas the post-intervention HgbA1c level averaged 7.81%. A paired t test showed that this pre–post difference of 1.03 percentage points was statistically significant (*p* = 0.0007). For BMI, there was an average decline of 0.78 from the pre-intervention mean value of 40.56 to the post-intervention mean value of 39.78 (*p* = 0.03). Among the 29 participants who agreed to participate in our follow-up measure of their HgbA1c status one year after the intervention, a paired t test showed that there was no significant difference between the post-JUMP measure and the follow-up measure (*p* = 0.808). There was no statistically significant difference between the HgbA1c level of those participating in the maintenance program and that of those not participating (post-intervention t test of between-group difference: *p* = 0.271; follow-up t test of between-group difference: *p* = 0.457). Our single-arm, pilot study of the three-week group intervention of self-empowerment shows promising results in glycemic control and weight loss. The short duration and small number of sessions expected could make it more feasible for implementation and dissemination as compared with popular intervention protocols that require much longer periods of attendance, if the effectiveness of this patient group-based self-empowerment approach can be further established by randomized controlled studies in the future.

## 1. Introduction

In 2015, there were approximately 29.1 million people in the United States with diabetes mellitus, around 9.3% of the population [1]. From 2009 to 2012, 37% of the US population aged 20 years or older (~86 million citizens) met the criteria for prediabetes [1]. Adults with diabetes were 1.8 times more likely to be hospitalized for a myocardial infarction and 1.5 times more likely to have a cerebral vascular accident than those without diabetes [1]. The International Diabetes Foundation reports that worldwide, more people die from diabetes and its complications annually than for HIV/AIDs, tuberculosis, and malaria combined [2].

Diabetes prevention and treatment bear substantial relevance for health disparities in the United States, as racial and ethnic minorities [3,4,5] and people with lower socioeconomic status are disproportionately affected by this disease [6,7]. 

As with other chronic diseases, controlling diabetes mellitus is difficult to accomplish if management sessions are limited to 15-minute office visits. Integrating psychosocial concepts such as patient self-efficacy, empowerment, and peer support into management interventions can improve diabetes control [8]. A review of new approaches to diabetes care identifies a range of patient-centered strategies, including shared decision-making, motivational interviewing, shared medical appointments (SMAs), and multidisciplinary team collaboration as potential positive diabetic management interventions [9]. SMAs, a patient-centered approach in diabetes care [10], have been shown to yield improvement in various outcomes for patients with diabetes (e.g., HgbA1c, patient satisfaction, and depression) when compared with the more traditional physician–patient office visits [10,11,12,13,14], even though the statistical significance varies across different studies. Another patient-centered strategy is motivational interviewing [15], a psychotherapeutic concept that focuses on enhancing patient’s intrinsic motivation to initiate behavior change and accomplish a desired goal. Motivational interviewing takes a more passive role and allows patients to guide themselves on the paths of problem-solving and deductive reasoning to achieve the outcomes they desire [16]. Reciprocal peer support is a tactic whereby patients with diabetes are paired up to generate encouragement, coaching, and accountability, and most of the transformations are independent of healthcare professionals’ direct intervention [17,18,19,20,21,22,23,24,25]. So far, however, it has been noted that statistically significant improvement in hemoglobin A1c is rare in patients involved in projects that mainly focus on reciprocal peer support [18,20,22,23]. On the other hand, this approach’s element of ‘diabetes-specific social capital’ is hypothesized to have potential for cultivating reciprocity, promoting cohesion, and enhancing solidarity to help counteract the risk factor of social isolation [25]. Given the possible link between social isolation stress and oxidative stress [26], an intervention that holds promise in addressing social isolation might have potential of reducing oxidative stress (a risk factor for diabetes complications [26]) among diabetes patients. 

In prenatal care, Centering Pregnancy has become a widely recognized, effective approach in a group dynamic [27]. Its framework is a replacement for traditional prenatal visits that permits patients to share health concerns, compare experiences with pregnancy, cultivate an avenue for education, and develop a climate of support, thus empowering each woman to respond proactively to her pregnancy [27]. When applied to diabetes management, centering models have been shown to be effective in the management of glycemic control among pregnant women [28,29]. 

Given the documented effectiveness of patient-centering models in controlling gestational diabetes mellitus (GDM), it is reasonable to infer that a similar model might prove successful for glycemic control among Type 2 diabetes patients as well. In this study, we examine the impact of a patient-centering self-empowerment intervention among diabetes patients, which uses multidisciplinary education, collaborative learning, peer support, and development of diabetes-specific social capital to improve glycemic control. 

## 2. Methods

With the Institutional Review Board’s approval from Prisma Health (#Pro00040212), our study participants were recruited from the type II diabetes patient population at Prisma Health-Upstate Internal Medicine Resident Clinic, an outpatient unit dedicated to serving low-income outpatients, to participate in our group session-based three-week intervention. The Internal Medicine Resident Clinic in Greenville, South Carolina provides primary care to a largely underserved population with a payer mix of approximately 40% Medicare, 20% Medicaid, and the rest self-pay/hospital sponsorship. Inclusion criteria were that patients had primary care established at the Internal Medicine Resident clinic and held the diagnosis of diabetes for longer than one year. In preparation for the launch, a motivating and uplifting title of the project was chosen (JUMP), care providers in the clinic were educated about the program, and promotional materials (posters and pamphlets) were placed in waiting areas and exam rooms. 

Each group intervention session involves one to two internal medicine resident physician facilitators, a clinical diabetic educator, and 5–10 patients with diabetes. Instead of being structured like a traditional class, each session had a framework facilitated by the resident with the majority of the discussion being patient-led. The primary goals were to provide a collaborative learning environment and create a support group atmosphere to encourage self-empowerment. To facilitate open discussions, the group sizes were capped at 10 participants, and family members were encouraged to attend.

The core program was comprised of three weekly sessions, each lasting about 2.5 h with a healthy lunch provided. The focus of the first week was on community building—group members took turns sharing their personal “diabetic story”, with group reflection on common fears and struggles with their disease. There was also a collaborative “diabetes 101” session during the first week to establish a foundation of knowledge about diabetes, including diagnostic criteria, common terminology, and complications. Participants were encouraged to share their knowledge, and the health professionals ensured accuracy, while expanding on different aspects and clarifying misconceptions.

The second week focused on healthy eating and exercise. Discussion centered around the American Diabetes Association “Plate Method” of healthy eating. In addition, patients were guided on the proper technique of reading food labels by the resident physician and registered dietitian. Rubberized faux food was distributed within the group, and the participants were encouraged to practice and counsel each other in creating balanced meals. One important aspect that was addressed during this session was how to buy healthy food on a limited income. For this, handouts on various strategies for bargain shopping were provided, and participants discussed strategies for shopping on a fixed budget.

Finally, week three consisted of participant-led discussion on common problems associated with diabetes (e.g., hypoglycemia), coping skills, and solutions. At the conclusion of the three-week program, participants were given certificates of graduation.

At the end of each weekly meeting, participants were challenged to make an attainable, focused, and measurable goal to strive towards. At the beginning of the following week’s session, participants were invited to share their successes and struggles with their weekly goal. 

A total of 35 type II diabetes patients participated in our pilot study. The primary endpoint of this study was glycemic control, by measuring patients’ hemoglobin A1c before initiating JUMP and 3 to 6 months after completion. HgbA1c data points were collected from the labs in the participants’ routine clinic visits. Pre-JUMP HgbA1c values were pulled from the patients’ most recently recorded labs. All of the 35 participants had their HgbA1c checked within the previous four months before starting JUMP. Post-intervention HgbA1c values were taken from the labs between three months and no more than six months after the conclusion of the last JUMP session. The other endpoint of our pilot study was body mass index (BMI), which was determined before and after the JUMP intervention. Due to the time and budget limits of this study as well as the concern for patient privacy in this small sample, no patient information such as age and gender was collected for analysis.

All graduates from the initial cycle of JUMP were then invited to attend monthly maintenance sessions that covered various topics related to diabetes. These additional gatherings include sessions such as grocery store tours. During these tours, participants can practice strategies on avoiding impulse buys, reading food labels, and obtaining the proper ingredients for a healthy meal on a fixed budget. Other session topics include techniques for insulin administration, strategies for proper foot care, modified yoga sessions for various body sizes, depression discussions, meal planning for economical healthy eating, and cooking demonstrations. Out of the 35 diabetes patients who finished the three-week intervention, 29 agreed to stay in the study beyond the post-JUMP HgbA1c assessment and had their HgbA1c readings measured one year after the JUMP intervention. However, out of the 29 diabetes patients who stayed in the study beyond the post-JUMP assessment, 16 chose to participate in the JUMP-Forward monthly maintenance program, while 13 chose not to participate in the maintenance program yet agreed to get their HgbA1c measured at the one-year follow-up measurement point. For the 16 who did participate in the monthly maintenance program, they had attended six to nine monthly sessions up to the point of one-year follow-up intervention.

We ran paired t tests of these JUMP participants’ HgbA1c and BMI values before and after the three-week JUMP intervention. We ran a paired t test comparing these JUMP participants’ HgbA1c values at the follow-up measurement and their HgbA1c post-intervention values. We then ran an independent t test comparing the HgbA1c changes between those who were in the maintenance program and those who were not. 

## 3. Results 

**Figure 1:**
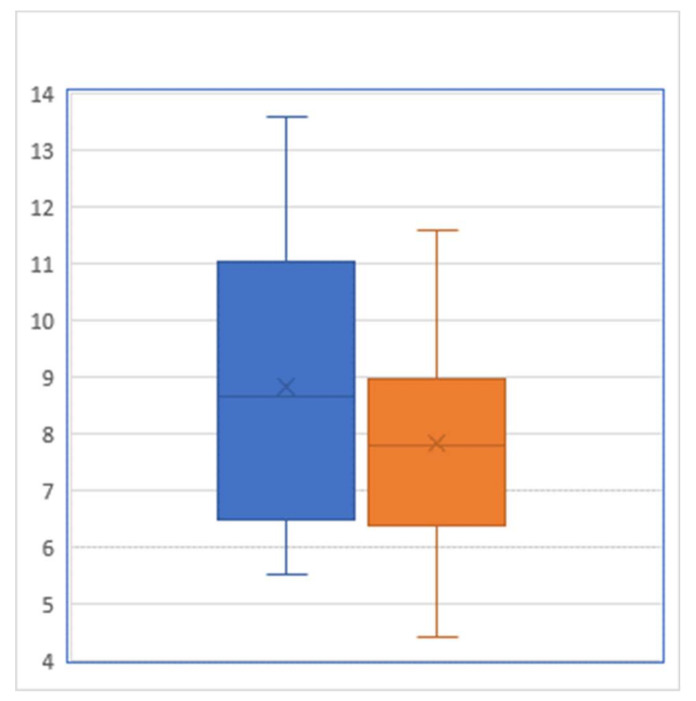
Box-and-whisker plot of hemoglobin A1C level (percent) before (left) and after (right) the intervention (Sample size = 29).

For the 35 participants of our JUMP intervention, the pre-intervention HgbA1c level averaged 8.84% with a standard deviation of 2.420%, whereas their post-intervention HgbA1c level averaged 7.81% with a standard deviation of 1.798% (Figure 1). A paired t test showed that this pre–post difference of 1.03 percentage points was statistically significant (*p* = 0.0007). From the viewpoint of controlling HgbA1c below 7%, there were 10 patients who reached this target prior to JUMP, while after the JUMP intervention, this number increased to 15: among the 25 patients who did not meet the target prior to the intervention, 4 successfully brought their HgbA1c down to reach the target level of <7%. 

**Figure 2:**
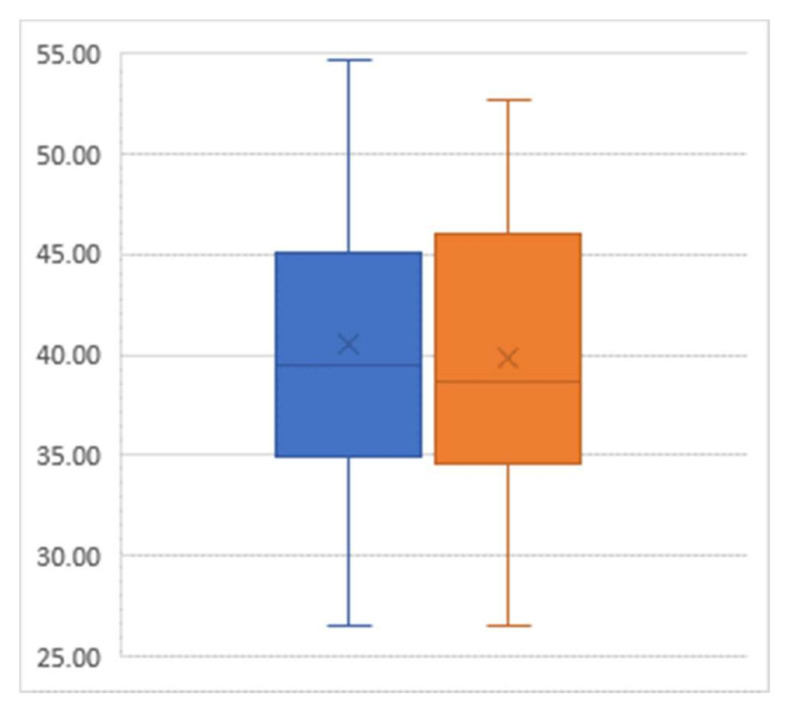
Box-and-whisker plot of body mass index (percent) pre-intervention (left) and post-intervention (right); Sample size = 29.

For the outcome of BMI, there was an average decline of 0.78 from the pre-intervention mean value of 40.56 to the post-intervention mean value of 39.78 (*p* = 0.03) (Figure 2). 

**Figure 3:**
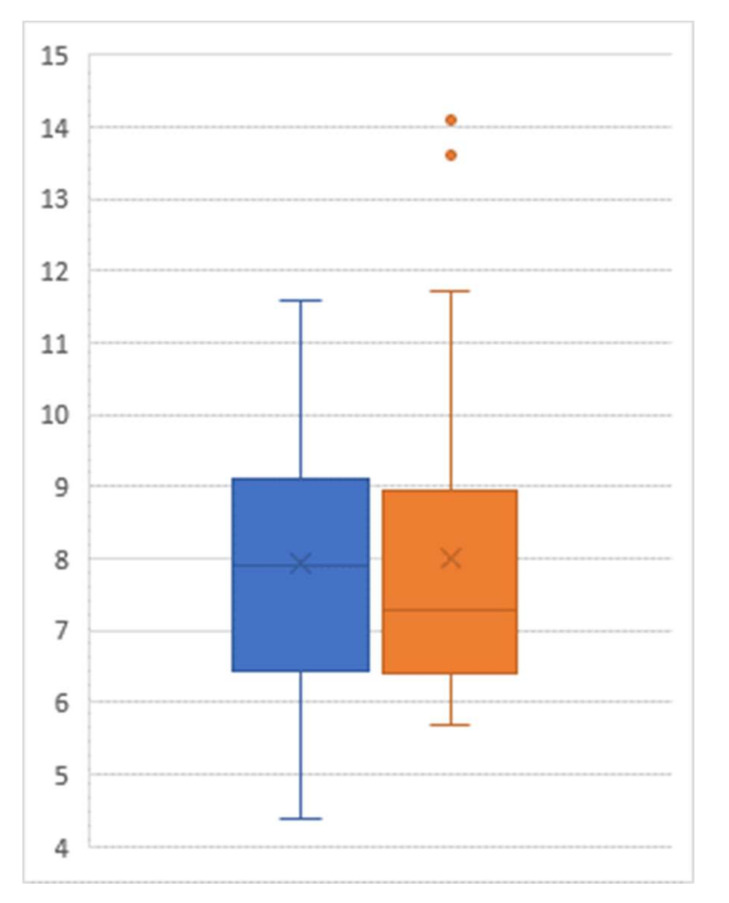
Box-and-whisker plot of hemoglobin A1C level (percent) post-intervention (left) and at follow-up (right); Sample size = 29.

Among the 29 participants who agreed to participate in our follow-up collection of their HgbA1C status one year after the post-JUMP measure, a paired t test showed that there was no significant difference between the post-JUMP measure and the follow-up measure (*p* = 0.808, Figure 3). There was no statistically significant difference between the HgbA1C values of those participating in the maintenance program and those not participating (post-JUMP t test of difference: *p* = 0.271; one-year follow-up t test of difference: *p* = 0.457).

## 4. Discussion

According to the World Health Organization, diabetes led to 1.5 million deaths in 2012, while a higher-than-optimal blood glucose level resulted in another 2.2 million deaths through elevating the risk of cardiovascular diseases and other diseases [30]. In addition, the medical cost for care of patients with diabetes is 2.3 times above that for those without diabetes [1]. This increased cost is a greater challenge to those with low socioeconomic status (SES), and those with low SES are more likely to develop diabetes [6,7].

Glycemic control is an important factor in assessing the impact of a diabetes management intervention on yielding alterations in further clinical outcomes. The United Kingdom Prospective Diabetes Study (UKPDS) found that the rate of microvascular complications from diabetes (e.g., retinopathy, nephropathy, and potentially neuropathy) fell by 25% when comparing patients with diabetes with a mean HgbA1c of 7.9% to those with a more tightly controlled mean HgbA1c at 7.0% [31]. Among those who successfully controlled their HgbA1c, diabetes-related deaths, myocardial infarctions, and all-cause mortality were significantly lower (25%, 18%, and 7% respectively) for each one percent improvement in HgbA1c [31]. Our pilot study is promising given that the mean decline in HgbA1c for JUMP participants was −1.03 percentage points (effectively, a decrease of 13.2%, *p*-value < 0.001), which compares favorably with the results of structured exercise programs that last at least 12 weeks (−0.67 percentage points, 95% Confidence Interval: −0.84, −0.23) [32]. 

The statistically significant BMI reduction in our study, which was effectively a 1.9% loss of body weight (a reduction of 0.78 from the pre-intervention BMI mean of 40.56), is smaller in size than the net decrease of 4.0% in body weight documented by the 2008 YMCA Diabetes Prevention Program study (DEPLOY) [33]. However, our group intervention approach only requires three weeks of intervention, which makes it easier for dissemination and implementation as compared with current intervention models expecting a longer duration of participation (e.g., the YMCA Diabetes Prevention Program’s approach, with 16 to 20 weeks of core curriculum delivery [33]). While our study is a single-arm pilot study with a limited sample size, our three-week self-empowerment group intervention model could have desirable cost effectiveness or cost-saving properties if the effect size can be maintained in follow-up randomized trials of this intervention approach. It is also encouraging to observe that the reduction in HgbA1c after JUMP was maintained among the participants whom we followed up. As we found no significant difference between those who attended our monthly maintenance program and those who did not, it looks likely that the maintenance program can be omitted from the overall JUMP intervention.

Our project has several limitations. First, our study is not a randomized experiment with a control group similar to the group that participated in our JUMP intervention. Thus, we cannot rule out the possibility that people who chose to participate in our intervention could be a highly motivated group that would accomplish HgbA1c reduction even without the intervention. Second, the sample size was modest, and so the number of participants would need to be expanded in the future to assess for consistent results; the lack of measurement about confounding factors further limits the generalizability of this study. Third, the timing of outcome measurement in this study was dependent on routine office visits with a primary care physician, which might or might not be the optimal timing for outcome assessment. Finally, we are aware that 6 out of 35 participants dropped out from the intervention and we were unable to track and report their key health outcome, which could create a selection bias that affects the results of this study.

Based upon our preliminary evidence from this pilot study, we will look to conduct a randomized controlled trial to further examine the effectiveness of this three-week self-empowerment group intervention. Future directions of JUMP also include ways to trend diabetes-specific measures via patient self-reporting questionnaires such as the diabetes empowerment scale [34] and the PHQ-9 [35] (given the common comorbidity of depression among diabetes patients [36]). Moreover, the participation of other members of a healthcare team, especially allied health professionals (nurse practitioners, physician assistants) and behavior health counselors, will further enhance and establish similar care innovations as part of the Patient-Centered Medical Home model [37]. 

By integrating the concepts of shared medical appointments [38], reciprocal peer support, motivational interviewing, and centering care into a three-week group intervention, our JUMP program shows that, through collaborative learning in a group setting, patients could improve their glycemic control with a relatively shorter time commitment for session attendance than that required by popular diabetes education program. Despite the preliminary nature of our study and the limits we described above, our finding provides clues for randomized controlled studies of patient self-empowerment group interventions for type 2 diabetes patients.
